# Comparative physical and mechanical properties of a 3D printed temporary crown and bridge restorative material

**DOI:** 10.4317/jced.60507

**Published:** 2023-06-01

**Authors:** Fabio Rizzante, Tamires Bueno, Genine Guimarães, Guilherme Moura, Sorin Teich, Adilson Furuse, Gustavo Mendonça

**Affiliations:** 1 DDS, MSc, PhD, MBA. Department of Oral Rehabilitation, James B. Edwards College of Dental Medicine – Medical University of South Carolina, Charleston, SC, USA; 2 DDS, MSc, PhD. CPO Dental Center, Bauru, SP, BrazilDDS, MSc, PhD. CPO Dental Center, Bauru, SP, Brazil; 3 DDS, MSc. Department of Operative Dentistry, Endodontics and Dental Materials, Bauru School of Dentistry - University of São Paulo, Bauru, SP, Brazil; 4 DDS, MSc, PhD. Department of Operative Dentistry and Dental Materials, School of Dentistry - Federal University of Uberlândia, Uberlândia, MG, Brazil; 5 DMD, MBA. Department of Oral Rehabilitation, James B. Edwards College of Dental Medicine – Medical University of South Carolina, Charleston, SC, USA; 6 DDS, MSc, PhD. Department of Operative Dentistry, Endodontics and Dental Materials, Bauru School of Dentistry - University of São Paulo, Bauru, SP, Brazil; 7 DDS, MSc, PhD. Department of General Practice, Virginia Commonwealth University School of Dentistry, Richmond, VA, USA

## Abstract

**Background:**

The objective was to compare physic-mechanical properties of different materials used for temporary restorations.

**Material and Methods:**

Protemp 4/bisacrylic resin, Jet/acrylic resin, and Nexdent C&B/3D-printed resin samples (10mm diameter x 2mm thickness) were analyzed for surface roughness and color stability tests (baseline, after 5 thousand brushing cycles; and after artificial aging in water at 60oC for 24 hours) and Knoop microhardness. All data were checked for normality using Shapiro-Wilk test. Surface roughness and color stability were analyzed using two-way repeated measurements ANOVA, microhardness data was subjected to one-way ANOVA. All tests were followed by Tukey test and were performed with α=0.05.

**Results:**

For roughness, material (*p*=.002), time points (*p*=.002) and interaction between both (*p*<.001) were significant. All groups presented similar roughness for measurements of baseline and after brushing. After artificial aging, 3D printed resin showed decreased roughness when compared with other resins, and with its baseline reading. Acrylic resin showed an increase in surface roughness (when compared with measurement after brushing cycles). Considering color stability, only the material (*p*=.039) and the time (*p*<0.001) were significant. All groups showed similar color variation before and after artificial aging. There was an increase in color alteration after artificial aging for all groups. Considering microhardness test (*p*<.001), the 3D printed resin showed the highest values and acrylic resin the lowest. Bysacylic resin was similar to both 3D printed and acrylic resins.

**Conclusions:**

The tested 3D printed resins present similar or better properties than other tested temporary materials while being integrated with the digital workflow.

** Key words:**Disinfection methods, hydroxyl radical, environment, surfaces, dentistry.

## Introduction

The use of temporaries in restorative dentistry consists in a very important step for assessment and communication of the final planned outcomes with patients and laboratory technicians. In addition, it is crucial for maintenance of the position, pulpal and periodontal health of the prepared teeth, as well as for maintenance of occlusal stability or diagnosis of occlusal reestablishment (1,2).

It is important for a temporary restoration to fully seat on the preparation, sealing all the margins, while presenting proximal and occlusal contact points to prevent alterations in tooth tridimensional position. Moreover, it should present low surface roughness since it may lead to bacterial adhesion, fungal infestation or facilitate biofilm formation and material discoloration (1,3); adequate surface hardness, which is related with resistance to abrasion, dimensional changes and maintenance of sTable occlusion and longevity of the appliance (2,4,5); as well as color stability for esthetic cases in which the temporaries will be used for a medium-long term.(2,4) Other desired properties for temporary materials include easy handling and possibility of being relined and repaired (1,2).

More recently, it also became highly desired that temporary restorations can be fully integrated with the digital workflow (1-3,6-10). Within this context, fast prototyping/3D printing has been introduced into the dental market, offering integration with the digital workflow using a potentially lower cost equipment (3D printer), reducing waste, and presenting similar fit when compared with conventional subtractive methods (2,8,10,11).

Several materials can be used for fabrication of temporary restorations, ranging from unfilled to filled resins, obtained from conventional, digital, or association of methods (2,11,12). Considering low-cost methods, the most common used materials are acrylic and bisacrylic resins. With the popularization of digital dentistry and 3D printing, fast prototyped temporaries might become a standard in the daily clinical activities, especially considering that acrylic and bisacrylic resins require more steps such as printing the model with the final treatment plan and creating a matrix/template in order to be integrated with the digital workflow, increasing clinical time and costs (12). Literature reports controversial properties for 3D printed resins when compared with traditional acrylic and bisacrylic resins (2,9,13). Moreover, there is limited information about the long-term properties of 3D printed parts, especially regarding surface roughness and color stability (3,10).

That being said, the purpose of the present study was to assess the surface microhardness, surface roughness and color stability of different resins used for temporary restorations. The tested null hypotheses were:

- there would be no differences on the surface roughness considering the tested materials and different timepoints

- there would be no differences on color stability considering the tested materials and different timepoints

- there would be no differences on microhardness considering the tested materials

## Material and Methods

The present study assessed different resins in 3 levels (Table 1) having as response variables the surface roughness (evaluated with a profilometer), surface microhardness (evaluated with a microhardness machine), and color stability (evaluated with a spectrophotometer).

**Table 1:**
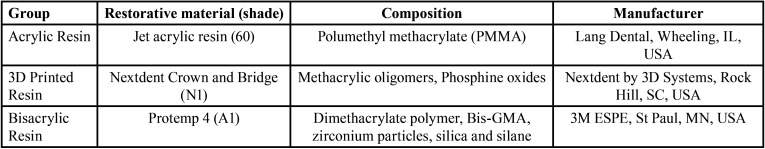
Different groups with respective composition and manufacturers.

Ten samples were made for each group using a bi-part Teflon mold (10mm diameter x 2mm height), except for the 3D printed resin group. For Protemp 4 and Jet resin groups, the samples were covered by a glass slide to standardize the surface and dimensions and left in the mold during 15 minutes for complete set.

The 3D printed resin samples (Nextdent C&B, 3D Systems, Rock Hill, SC, USA) were designed on an open CAD software (Meshmixer v.3.5.474, Autodesk, San Rafael, CA, USA), exported as stereolithography files (.STL), and printed using a stereolithography-based (SLA) 3D printer (Form 2, Formlabs, Somerville, MA, USA) using preform software (v.3.1.2). All samples were printed at 90º with supports set to a density of 1, point size of 600 μm, and layer thickness of 100μm, with the resin parameter set to “white” (7). The 3D printed samples were detached from the building platform, washed in isopropyl alcohol at 99.5% using a Formwash (Formlabs) during 20 minutes, followed by post polymerization using the Formcure (Formlabs) during 30 minutes at 80ºC, immersed in glycerin.

All samples were polished using a sequence of polishing discs (sof-lex, 3M ESPE, St Paul, MN, USA), from the coarsest (red) up to the smoothest (yellow), during 10s for each sample, per disc. The discs were changed for every 3 specimens and between each polishing step, samples were immersed in an ultrasonic bath during 5 minutes to remove debris. Each specimen was used for all the tests (surface roughness, microhardness and color stability).

-Surface Roughness

Every specimen was assessed for roughness using a profilometer (Hommel Tester T1000- Hommelwerke GmbH, Villingen-Schwenningen, Germany), set for 5µm, 90º, 1.6mN. Each specimen’s surface was read 3 times and the average value was considered as surface roughness.

The readings were performed at three time points: Baseline (R1), after abrasion test (R2), and after artificial aging (R3). Following the surface roughness test, samples were stored in a dark, dry storage at 37ºC for 24 hours.

-Color stability

The color stability test was performed immediately after polishing (baseline 1/R1), after abrasion test (R2), and after artificial aging (24 hours immersion in water at 60ºC/ R3). A CIE-lab based colorimeter was used (Easyshade, Vita Zahnfabrik, Bad Säckingen, Germany). The colorimeter was calibrated before each reading time point (R1, R2, and R3). After removing the humidity from specimens’ surface using absorbent paper, resin discs were placed over a white sheet (to standardize the background), the probe was handheld perpendicular to the specimen and 3 readings were performed observing homogeneity of results. The average of the 3 readings were considered as the final color of each specimen (14,15).

**Figure 1:**
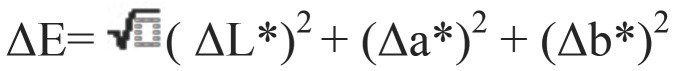
Formula.

The color change was calculated with the following formula: (Fig. 1).

In which ΔL*, Δa* and Δb* corresponds to the color differences observed between the baseline and the subsequent measurements.

-Microhardness

Specimens were evaluated for Knoop microhardness using a microhardness tester (Micromet 6040, Buehler, Lake Bluff, IL, EUA) 24 hours after the R1 reading for surface roughness. Every specimen’s surface was assessed through 3 readings separated by 0.4mm, with crosshead speed of 0.5mm/min, and 25g force during 5s. The longest diagonal in the resulting impression was evaluated using the built-in stereomicroscope at 10X magnification, and each impression result was determined by automatic calculation. The mean value of the three readings was considered as the final value for each specimen.

-Abrasion test and artificial aging

Following the microhardness test, the specimens of each group (n=10) were mounted on a brushing machine (Elquip, Sao Carlos, SP, Brazil) and 5000 brushing cycles (20mm amplitude and 300g load) were performed (simulating 6 months) using a tooth brush with soft bristles (Colgate Classic Clean, Colgate-Palmolive Co., New York, NY, USA) and toothpaste with RDH = 70 (Colgate Maximum Protection, Colgate-Palmolive Co.). All the cycles were performed at 37 ± 1ºC and 4.5 cycles/s.(16) Every 2 minutes, 0.4mL of slurry (toothpaste and distilled water at a ratio of 1:2) was automatically injected and the toothbrushes were changed after every 5000 brushing cycles. After brushing, specimens were stored immersed in water at 60ºC in absence of light during 24 hours (14,15).

-Statistical analysis

All data were evaluated for homogeneity using Shapiro-Wilk test. Surface Roughness and Color stability results were assessed using two-way repeated measurements ANOVA. Microhardness results were evaluated using one-way ANOVA. All analyses of variance tests were followed by Tukey’s test. All statistical analyses had 5% as significance level.

## Results

Considering roughness (Table 2), the material (*p*=.002), the different time points (*p*=.002) and the interaction between material and time (*p*<.001) were significant. For material, printed resin<acrylic resin=bisacrylic resin. For timepoints, R2<R1=R3. It was possible to observe that all groups showed similar roughness for baseline and after brushing. After immersion in water at 60ºC for 24 hours, the 3D printed resin showed decreased roughness when compared with other resins, and when compared with its baseline reading. Acrylic resin showed an increase in surface roughness (when compared with measurement after brushing cycles).

**Table 2:**
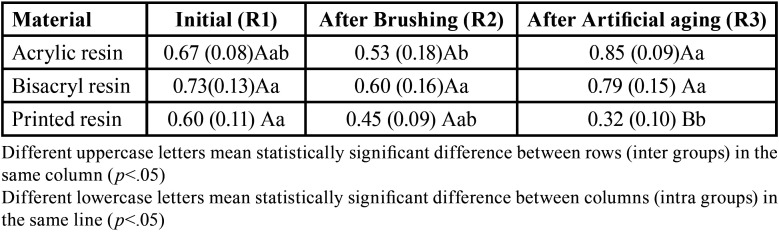
Roughness (Ra) of the tested material at the different timepoints.

**Figure 2:**
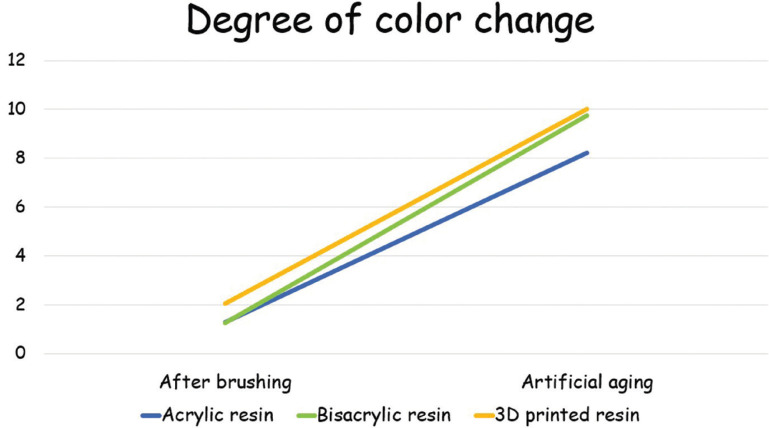
Degree of color change (ΔE) for different tested materials.

Considering color stability (Fig. 2), only the material (*p*=.039) and the time (*p*<0.001) were significant. For material, acrylic resin=bisacrylic resin, and 3D printed resin showed similar color alteration to bisacrylic resin but higher than acrylic resin. For time, artificial aging>abrasion test. All groups showed similar color alteration before and after artificial aging. Nevertheless, there was an increase in color alteration after artificial aging in water at 60ºC during 24 hours for all groups.

**Figure 3:**
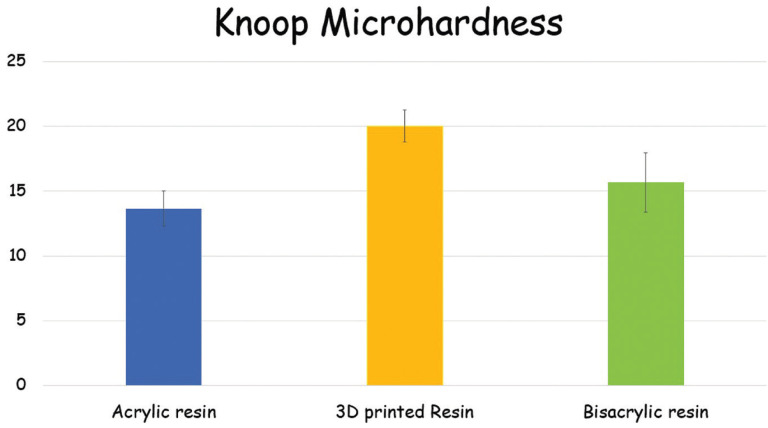
Knoop microhardness (KHN) of tested materials.

For microhardness (Fig. 3), different materials showed different results (*p*<.001). The 3D printed resin showed higher values than acrylic resin. Bisacrylic resin showed similar microhardness as 3D printed and acrylic resins.

## Discussion

The use of 3D printed temporary restorations, with its potential increase in productivity and predictability explain its quick adoption and development within practices and dental schools (1-3,6-10). Nevertheless, there is limited information about long-term properties of 3D printed resins compared to conventional materials used for temporary restorations.

Considering the results of the present study, all null hypotheses were rejected and overall results reflect the materials composition as previously described (9). Both Protemp 4 and Jet resins are self-curing resins. Protemp 4 is based on bisacrylic resins with multifunctional monomers (such as Bis-GMA or TEGDMA), which increases the mechanical properties of the material, especially with the association of filler particles (7). Jet is primarily composed of methyl methacrylate without fillers, which results in a weaker and more hydrophilic monomer when compared to bisacrylic resin (8). In addition to the materials composition, there is also a concern about incorporation of defects such as air bubbles and porosities due to manual or automixing manipulation, which can jeopardize the materials properties when compared with 3D printed materials (17).

The 3D printed resin used in the present study is also unfilled, having at least 90% methacrylic oligomers and up to 3% phosphine oxides as photoinitiators in the monomer blend (2,7). It is noteworthy the concern about the final properties of 3D printed parts as they are influenced not only by the materials composition, but also by printing parameters, orientation, and post-processing methods, which may increase degree of conversion and physicomechanical properties (2,8,9,18).

There is an increasing concern regarding the surface properties of 3D printed resins as surface roughness is correlated with biofilm accumulation and color stability. Moreover, accumulation of plaque around temporary restorations may result in tissues inflammation and jeopardize final restorations (3,10). The initial surface roughness was similar for all groups showing that the polishing procedures resulted in a standardized surface. Although only significant for the acrylic resin group, a tendency of reduction in surface roughness was observed after brushing simulation probably due to a polishing effect caused by the brushes associated with the toothpaste in a continuous, repetitive and standardized movement, removing possible irregularities from the samples surface, or filling micro spaces with debris (19). After artificial aging, acrylic and bisacrylic resins showed the highest roughness (similar to the initial reading), while the 3D printed resin showed the lowest results. It is noteworthy the significant reduction in surface roughness for the 3D printed resin after artificial aging, which may be explained by a relatively more hydrophobic monomer when compared to acrylic and bisacrylic resins, contributing to “wash out” remaining particles and micro spaces present on the surface after the brushing simulation (8).

Although the comparison between surface roughness of 3D printed and conventional temporary materials are controversial and largely dependent on composition of tested resin and printing orientation (18,20), this study results are in agreement with the literature (21). The polishing protocol used for the 3D printed parts may have contributed to an initial roughness similar to the other tested materials as there are suggestions for polishing and using surface glaze/sealant materials to improve surface roughness and color stability of 3D printed resins (10,22). Nevertheless, all samples showed roughness higher than the threshold for plaque accumulation (0.2 μm) (10,23).

The color stability was similar for all tested materials for both timepoints, and there was an increase in color alteration after artificial aging. Literature is controversial about color stability for 3D printed parts, showing better (24) or worse (20) color stability when compared to traditional temporary resins. This may be explained by the standardized surface polishing protocol, resulting in similar initial surface roughness. The increase in color alteration after artificial aging is compatible with the literature regarding color stability of polymeric materials (14,15,18,20,21,24,25). and higher than the clinical threshold of 3.3 (14,15). The low (bisacryl resin) or absence (printed and acrylic resins) of filler content might have contributed with a less sTable polymer matrix, explaining the present results (7). Moreover, the poor color stability of 3D printed resins has been attributed to the higher hydrophilicity/polarity of the polymer, a lack of filler particles, increased surface roughness, presence of residual monomers, high solubility, among others, being dependent on the material and post processing protocols (20,22,24,25).

Microhardness is an important predictor of the abrasion resistance of a material. Acrylic resin showed the lowest values, although similar to the bisacryl resin, while the 3D printed resin showed the highest values (yet similar to bisacrylic resin). Such results are in agreement with the literature reporting better resistance to abrasion for 3D printed resins when compared with acrylic resins (4,13).

This study focused in assessing the properties of low-cost temporary materials. Thus, only one material for each class (acrylic, bisacrylic, and 3D printed resins) was selected and different products and protocols may show different results. Nevertheless, based on the results of the present study, the 3D printed resin can be considered as an adequate low-cost temporary material, showing similar or better properties than acrylic and bisacrylic resins, and having as advantages the fully integration with the digital workflow and lower technical sensitivity.

All samples were printed with a 90° as it has been reported to result in the best mechanical properties and requires less supports and post-processing (such as trimming and polishing), preventing creation of defects on the surface and/or decrease in mechanical properties (5,7,18). Future studies should focus on the effects of different equipment and parameters in the final properties of 3D printed parts, in order to better explains the controversial results in the literature.

One may ask about temporary blocks for CAD-CAM and, although they are also integrated with the digital workflow, the equipment and material are far more expensive than some 3D printers and 3D resins available in the market, and present limitations related to shaping complex details and manufacturing speed when long span temporaries are needed (2).

## Conclusions

The tested 3D printed resins present similar or better surface roughness, color stability and surface microhardness than other temporary materials (i.e. acrylic resin and bisacylic resin), while providing the advantages and versatility of a completely digital workflow.
